# Mental Health Outcomes Amongst Health Care Workers During COVID 19 Pandemic in Saudi Arabia

**DOI:** 10.3389/fpsyt.2020.619540

**Published:** 2021-01-14

**Authors:** Maha Al Ammari, Khizra Sultana, Abin Thomas, Lolowa Al Swaidan, Nouf Al Harthi

**Affiliations:** ^1^Department of Pharmacy Service, King Abdul Aziz Medical City, Riyadh, Saudi Arabia; ^2^Ministry of National Guard Health Affairs (MNGHA), King Saud Bin Abdulaziz University for Health Sciences (KSAU-HS), Riyadh, Saudi Arabia; ^3^King Abdullah International Medical Research Center (KAIMRC), Riyadh, Saudi Arabia; ^4^King Saud Bin Abdulaziz University for Health Sciences (KSAU-HS), Riyadh, Saudi Arabia; ^5^College of Biomedical and Life Sciences, Cardiff University, Cardiff, United Kingdom; ^6^Centre for Trials Research, Cardiff University, Cardiff, United Kingdom

**Keywords:** mental health, Saudi Arabia (KSA), COVID-19, outcomes—health care, health care workers

## Abstract

**Objectives:** The study aimed to assess the mental health outcomes and associated factors among health care workers during COVID 19 in Saudi Arabia.

**Design, Setting, and Participants:** We conducted a cross-sectional survey of health care workers from tertiary care and ministry of health Centers across the Central, Eastern, and Western regions of Saudi Arabia. There were 1,130 participants in the survey, and we collected demographic and mental health measurements from the participants.

**Primary Outcomes and Measures:** The magnitude of symptoms of depression, anxiety, and insomnia was measured using the original version of 9-item patient health questionnaire (PHQ-9), the 7-item generalized anxiety disorder scale (GAD-7), and 7-item insomnia severity index (ISI). We use the multiple logistic regression analysis to identify the associated risk factors of individual outcomes.

**Results:** The scores on the PHQ-9 showed that the largest proportion of health care workers (76.93%) experienced only normal to mild depression (50.83 and 26.1%, respectively). The scores on the GAD-7 showed that the largest proportion of health care workers (78.88%) experienced minimal to mild anxiety (50.41 and 28.47%, respectively). The scores on the ISI showed that the largest proportion of health care workers (85.83%) experienced absence to subthreshold insomnia (57.08 and 28.75%, respectively). The risk factors for depression in health care workers were Saudi, living with family, working from an isolated room at home and frontline worker. For anxiety, being female was risk factor and for insomnia, being frontline worker was risk factor.

**Conclusion:** It was observed that the symptoms of depression, anxiety, and insomnia were reported in a lower proportion of health care workers in our study. The participants who were female, frontline workers, Saudi, living with family, and working from home in isolated rooms were predisposed to developing psychological disorders.

## Introduction

The *severe acute respiratory syndrome coronavirus 2* (SARS-CoV-2), also known as COVID-19, started in Wuhan's Chinese city at the end of December 2019 and was declared a global public health concern by WHO at the end of January 2020 (1). As of 14 May 2020, the Coronavirus positive cases were at 44,830 in the Kingdom of Saudi Arabia (KSA), which was the highest in the Gulf Cooperation Council states (2). In the context of the COVID-19 pandemic, it is essential to evaluate the mental health of the Health Care Workers (HCWs) as they are constantly exposing themselves to the risk of infection and separating themselves from their families for weeks to avoid transmitting the virus to them. HCWs are also most susceptible to emotional distress in the current pandemic due to their risk of exposure to the virus, fear about infecting and caring for their loved ones, scarcity of personal protective equipment (PPE), and long work hours (3, 4). During the 2003 SARS outbreak, a study reported that HCWs feared infecting their families or friends and felt stigmatized because they were known to contact with sick patients (5–8) This led to them experiencing significant long-term psychological stress even after 1 year from the outbreak (8). It has been observed that in Italy and the US, where this pandemic took a toll on the health care system, in addition to a shortage of infectious disease consultants, other doctors were trained to care for an influx of patients within 7 days (9). Many of them would have post-traumatic stress disorder or other mental health problems down the line as they have no experience watching a patient being intubated or die in front of them (9).

Health care workers already deal with disproportionately high rates of depression—about three times higher than the general public (10). However, the strain of treating coronavirus patients, and the impossible decisions many doctors and nurses are being forced to make, will likely worsen their mental health (11). Several studies were done in KSA during the MERS-CoV outbreak (12–16). One of the studies reported that half of the respondents for MERS-CoV reported decreased work performance, and 75% reported having psychological problems. In this study, 61.2% of HCW reported anxiety about contracting MERS-CoV from patients (12). None of the surveys done during this period used validated instruments to assess the mental well-being of the HCWs.

The workplace is a vital setting for activities to improve well-being for adults. By addressing mental health issues in the workplace, employers can reduce healthcare costs for their businesses and employees. Evaluation and intervention for psychosocial concerns must be undertaken in these settings. Poor mental health and stress negatively affect the employees by decreasing job performance, engagement in work, communication with coworkers, and physical capabilities (17). Protecting and maintaining the mental well-being of health care workers in KSA was a priority during the COVID19 pandemic. It is crucial not only to assess the mental health of HCWs but also associated factors or risk factors as these factors predict the probability of developing the condition, which is not recognized by the patient (18). A risk factor is any attribute, characteristic, or exposure of an individual that increases the likelihood of developing a disease (19).

Several studies have explored the psychological impact of COVID 19 in HCWs and the general public using different scales in KSA. Alateeq et al. conducted a study using the Patient Health Questionnaire (PHQ-9) and Generalized Anxiety Disorder (GAD-7) in the ministry of health care hospitals, mostly located in the Qassim region. The authors used the Arabic version of these questionnaires and in only Arabic speaking population (20). Al Mater et al. conducted their study on the psychological health of ophthalmologists from different regions in KSA. They used the English version of PHQ-9, GAD-7, Insomnia Severity Index (ISI), and Perceived Stress Scale (PSS) (21). Alzaid et al. administered a survey in the Eastern Province of KSA in the English language to study anxiety in HCWs. The authors developed the survey with 34 questions, divided into four sections, and the last section included the GAD-7 scale to measure anxiety (22). Zaki et al. surveyed the Northen armed forces hospital in Arabic and English to report the stress and psychological consequences in HCWs. The authors designed the instrument with four sections and the last section included the Impact of Events Scale-Revised(IES-R) (23). Alenazi et al. (24) surveyed the HCWs' in the Arabic language to study the prevalence and predictors of anxiety in 13 regions of KSA. Alsulais et al. explored the psychological impact of COVID 19 in physicians. The authors adapted a questionnaire in the English language from a previous Canadian study done on SARS (25). Temsah et al. surveyed HCWs in King Khalid University Hospital Riyadh. The authors designed the survey and included the GAD-7 to assess anxiety (26). Al Hanawi et al. (27) conducted their study using the Peritraumatic Distress Index (CPDI) administered in Arabic in HCWs and the general public.

In this study, we assessed the mental health and associated risk factors among HCWs during the COVID 19 pandemic using the English version of validated assessment scales—Patient Health Questionnaire (PHQ-9), Generalized Anxiety Disorder (GAD-7), and Insomnia Severity Index (ISI) scale to measure the depression, anxiety, and insomnia.

## Methods

This study is a cross-sectional web-based survey administered using LIME software for HCWs from 27 April to 4 May 2020. We used the purposive sampling method for this study. The weblink to the questionnaire was sent to prospective participants through WhatsApp, Twitter, and official emails. The number of HCWs who participated in the survey was 1,130, out of which 720 completed the survey. All participants were given information about the purpose of the study and were assured confidentiality. Participating in the survey indicated their consent to the study. The participants included health care providers (physicians, nurses, pharmacists, respiratory therapists, physical therapists, nutritionist, and paramedics) working in different departments in Ministry of National Guard Health Affairs (MNGHA) hospitals across the Central, Eastern, and Western regions and some Ministry of Health (MOH) Centers across Central Region.

### Outcomes and Covariates

We assessed the level of depression, anxiety, and insomnia using the validated scales original version. The 9-item Patient Health Questionnaire(PHQ-9; range, 0–27) the 7-item Generalized Anxiety Disorder (GAD-7; range, 0–21) scale, and the 7-item Insomnia Severity Index (ISI; range, 0–28) (28–30).

The PHQ-9 helps in screening, diagnosing, monitoring, and measuring the severity of depression. It has a one factorial structure with nine questions. These questions are scored 0–3 (“Not at all,” “Several days,” “More than half the days,” and “Nearly every day”), providing a 0 to 27 severity score. There is an additional question in the end: a follow-up non-scored question that screens and assigns weight to the degree to which depressive problems have affected the patient's level of function. The total PHQ-9 score is classified into 0–4 = “Minimal depression,” 5–9 = “Mild depression,” 10–14 = “Moderate depression,” 15–19 = “Moderately severe depression,” and 20–27 = “Severe depression” (29).

The GAD-7 helps in screening, diagnosing, and measuring the severity of anxiety. It consists of one factorial structure with seven questions. These questions are scored 0–3 (“not at all,” “several days,” “more than half the days,” and “nearly every day”) providing 0–21 severity score. There is an additional question in the end, which is a follow-up non-scored question that assigns weight to the degree to which anxiety problems have affected the patient's functional level. The GAD-7 score is classified into 0–4 = “Minimal anxiety,” 5–9 = “Mild anxiety,” 10–14 = “Moderate anxiety,” and 15–21 = “Severe anxiety” (31).

The Insomnia Severity Index (ISI) is a brief instrument that assesses the severity of both nighttime and daytime insomnia components. It comprises of one factorial structure with seven questions. A 5 point Likert scale is used to rate each item from 0 to 4 with different answer options according to the questions providing 0 to 28 severity scores. The ISI score is classified into 0–7 = “No clinically significant insomnia,” 8–14 = “Subthreshold insomnia,” 15–21 = “Clinical insomnia (moderate severity),” and 22–28 = “Clinical insomnia (severe)” (28).

Demographic data was self-reported by the participants which included “age,” “gender,” “nationality,” “region,” “marital status,” “educational level,” “have children,” and “living with family during the outbreak.” Job-related questions include “department,” “profession,” “clinical experience,” “working position (front line or second line),” “duration of employment in MNGHA.”

### Analysis

The data was extracted in MS Excel, and incomplete or missing responses were removed; hence the analysis data was complete without any missing values. Data analysis was performed using SAS 9.4 [Copyright (c) 2016 by SAS Institute Inc., Cary, NC, USA]. The PHQ-9, GAD-7, and ISI questions were scored as described in the guidelines (28–30). Due to fewer frequencies, some multicategory variables were merged into lesser categories such as Central and Other regions; Marital status: Single and Married; Departments: Critical care, Pharmacy, Medicine, and Others; Professional title: Physician, Nurse, Pharmacist, and Others.

The tools' scores were presented as medians, and interquartile ranges (IQRs) and frequency and percentages were used to summarize the demographics, work environment, and the living condition related questions. The analysis estimated the relative frequencies of demographic, work-related, and living condition related variables across the PHQ-9, GAD-7, and ISI categories. A Chi-square test is used to calculate the *p*-value for all cross-tabulation. A two tailed test with *p* < 0.05 is considered as evidence for an association.

Multiple logistic regression models are used to determine the factors associated with symptoms of “Moderate or Severe depression,” “Moderate or Severe anxiety,” and “Subthreshold, Moderate or Severe insomnia.” All variables with a *p*-value of < 0.05 in the bivariate analysis are selected for the logistic regression models. For modeling the “Moderate or Severe depression,” the gender, age, nationality, experience, belong to ministry or not, marital status, living with family, have children, and work position variables are selected as independent variables. For modeling the “Moderate or Severe anxiety,” the gender, age, nationality, profession, experience, living with family, and work position variables are independent variables. For modeling the “Subthreshold, Moderate or Severe insomnia,” gender, age, experience, and work position are used as independent variables. The multivariate results are reported as adjusted odds ratios and corresponding 95% confidence limits.

#### Ethics

The study was planned and conducted as per the declaration of Helsinki 1964 and is approved by King Abdullah International Medical Research Center (KAIMRC) ethics review board with protocol no RC20/169/R.

## Results

### Demographics

In this study, 1,130 HCWs participated, with 720 complete responses. Of the respondents, 194 (26.94%) Physicians, 262 (36.39%) Nurses, and 171 (23.75%) pharmacists completed the survey. The participants' female respondents (64.17%) were almost double the males (35.83%), with nearly 75% above 30 years of age. Saudis (57.22%) were slightly higher compared to expatriates, with the majority of the participants working in the central region (84.2%) in tertiary care hospitals (71.77%). Most of the participants were postgraduates (91.81%), with nearly 60% with >10 years of professional experience. A little over 60% were single, and 61% were working from home. Nearly 60% of the participants had children, and 62% had no previous experience with the pandemic, and only 32.5% have received some training to work during such a crisis. Only about one-third of the participants were frontline HCWs directly engaged in diagnosing, treating, or caring for the patients with suspected COVID-19 (Table 1).

**Table 1:** Demographics.

**Overall** ***N*** **(%)**		**720 (100)**	
**Gender**		**Number of years of professional**	
Male	258 (35.83)	1 to 3	96 (13.33)
Female	462 (64.17)	4 to 6	112 (15.56)
**Age**		7 to 9	92 (12.78)
18–25	31 (4.31)	>10	420 (58.33)
26–30	150 (20.83)	**Do you belong to the MNGHA**	
31–40	278 (38.61)	No	171 (23.75)
>40	261 (36.25)	Yes	549 (76.25)
**Nationality**		**Type of hospital**	
Saudi	412 (57.22)	Secondary	125 (17.39)
Non-Saudi	308 (42.78)	Tertiary	516 (71.77)
**Region**		Other	78 (10.85)
Central	606 (84.2)	Missing	1
Western	67 (9.31)	**Marital Status**	
Eastern	30 (4.17)	Single	450 (62.5)
Other	17 (2.36)	Married	253 (35.14)
**Department**		Other	17 (2.36)
Critical care	156 (21.67)	**Are you living with your family**	
Emergency medicine	19 (2.64)	Yes, working from home	256 (35.56)
Surgical wards	22 (3.06)	No	280 (38.89)
Laboratory	15 (2.08)	Yes, isolated in separate room	184 (25.56)
Pharmacy	163 (22.64)	**Do you have children**	
Medicine	91 (12.64)	Yes	420 (58.33)
Other	254 (35.28)	No	300 (41.67)
**Professional title**		**Previous experience with pandemic**	
Physician	194 (26.94)	No	446 (61.94)
Nurse	262 (36.39)	Other	274 (38.06)
Respiratory therapist	11 (1.53)	**Any kind of training for pandemic**	
Pharmacist	171 (23.75)	No	486 (67.5)
Lab Technician	8 (1.11)	Other	234 (32.5)
Other	74 (10.2)	**What is your working position**	
		Front line	200 (27.78)
**Educational level**		Second line	520 (72.22)
Under graduate	59 (8.19)		
Post graduate	661 (91.81)		

### Scores of Measurement and Associated Factors

Table 2 reports the participants' overall responses, the median, interquartile range, and the Chi-square analysis. The median total score with IQR reported for depression was 4 (2.00–9.00). The scores on the PHQ-9 showed that the largest proportion of HCWs (76.93%) experienced normal to mild depression (50.83% and 26.1%, respectively). The rest (22.99%) reported moderate, moderately severe, and severe depression (13%, 7.91%, and 2.08%, respectively). The median total score with IQR reported for generalized anxiety was 4 (1.00–8.00). The scores on the GAD-7 showed that the largest proportion of HCWs (78.88%) experienced minimal to mild anxiety (50.41% and 28.47%, respectively). The rest (21.1%) reported moderate to severe anxiety (12.77% and 8.33%, respectively). The median total score with IQR reported for insomnia was 6 (2.00–12.00). The scores on the ISI showed that the largest proportion of HCWs (85.83%) experienced absence to subthreshold insomnia (57.08% and 28.75%, respectively). The rest (14.16%) reported moderately severe to severe insomnia (10.41% and 3.75%, respectively).

**Table 2:** Severity categories of depression, anxiety, and insomnia in total cohort and subgroups.

		**PHQ-9 depression symptoms**					**GAD-7 anxiety**				**ISI-insomnia symptoms**			
**Total score, median (IQR)**		**4.00 (2.00–9.00)**					**4.00 (1.00–8.00)**				**6.00 (2.00–12.00)**			
		**Normal**	**Mild**	**Moderate**	**Moderate Severe**	**Severe**	**Normal**	**Mild**	**Moderate**	**Severe**	**Absence**	**Sub threshold**	**Moderate Severity**	**Severe**
Total *N* (%)		366 (50.83)	188 (26.1)	94 (13)	57 (7.91)	15 (2.08)	363 (50.41)	205 (28.47)	92 (12.77)	60 (8.33)	411 (57.08)	207 (28.75)	75 (10.41)	27 (3.75)
**Age** ***N*** **(%)**	18–25	17 (54.84)	4 (12.9)	6 (19.35)	3 (9.68)	1 (3.23)	15 (48.39)	8 (25.81)	5 (16.13)	3 (9.68)	18 (58.06)	10 (32.26)	2 (6.45)	1 (3.23)
	26–30	50 (33.33)	52 (34.67)	32 (21.33)	14 (9.33)	2 (1.33)	63 (42)	44 (29.33)	25 (16.67)	18 (12)	67 (44.67)	57 (38)	20 (13.33)	6 (4)
	31–40	134 (48.2)	74 (26.62)	39 (14.03)	23 (8.27)	8 (2.88)	115 (41.37)	97 (34.89)	44 (15.83)	22 (7.91)	152 (54.68)	79 (28.42)	32 (11.51)	15 (5.4)
	>40	165 (63.22)	58 (22.22)	17 (6.51)	17 (6.51)	4 (1.53)	170 (65.13)	56 (21.46)	18 (6.9)	17 (6.51)	174 (66.67)	61 (23.37)	21 (8.05)	5 (1.92)
		**<0.0001**				**<0.0001**				**<0.0001**				
**Nationality No. (%)**	Saudi	178 (43.2)	123 (29.85)	60 (14.56)	39 (9.47)	12 (2.91)	181 (43.93)	127 (30.83)	62 (15.05)	42 (10.19)	221 (53.64)	128 (31.07)	45 (10.92)	18 (4.37)
	Non-Saudi	188 (61.04)	65 (21.1)	34 (11.04)	18 (5.84)	3 (0.97)	182 (59.09)	78 (25.32)	30 (9.74)	18 (5.84)	190 (61.69)	79 (25.65)	30 (9.74)	9 (2.92)
		**<0.0001**				**<0.0005**				0.171				
**Professional title** ***N*** **(%)**	Physician	92 (47.42)	51 (26.29)	25 (12.89)	21 (10.82)	5 (2.58)	100 (51.55)	43 (22.16)	26 (13.4)	25 (12.89)	115 (59.28)	45 (23.2)	28 (14.43)	6 (3.09)
	Nurse	156 (59.54)	54 (20.61)	32 (12.21)	18 (6.87)	2 (0.76)	151 (57.63)	72 (27.48)	26 (9.92)	13 (4.96)	152 (58.02)	74 (28.24)	25 (9.54)	11 (4.2)
	Pharmacist	74 (43.27)	56 (32.75)	23 (13.45)	13 (7.6)	5 (2.92)	73 (42.69)	60 (35.09)	23 (13.45)	15 (8.77)	93 (54.39)	60 (35.09)	13 (7.6)	5 (2.92)
	Other	44 (47.31)	27 (29.03)	14 (15.05)	5 (5.38)	3 (3.23)	39 (41.94)	30 (32.26)	17 (18.28)	7 (7.53)	51 (54.84)	28 (30.11)	9 (9.68)	5 (5.38)
		0.068				**0.004**				0.281				
**Belong to the Ministry** ***N*** **(%)**	Yes	290 (44.44)	128 (23.32)	75 (13.66)	45 (8.2)	11 (2)	291 (53.01)	149 (27.14)	65 (11.8)	44 (8.01)	312 (56.83)	157 (28.6)	58 (10.56)	22 (4.01)
	No	76 (44.44)	60 (35.06)	19 (11.11)	12 (7.02)	4 (2.34)	72 (42.11)	56 (32.75)	27 (15.8)	16 (9.36)	99 (57.89)	50 (29.24)	17 (9.94)	5 (2.92)
		**0.046**				0.094				0.919				
**Living with family** ***N*** **(%)**	Yes, working from home	137 (53.52)	68 (26.56)	26 (10.16)	19 (7.42)	6 (2.34)	132 (51.56)	73 (28.52)	31 (12.11)	20 (7.81)	155 (60.55)	64 (25)	29 (11.33)	8 (3.13)
	Yes working from home isolated room	76 (41.3)	49 (26.63)	37 (20.11)	16 (8.7)	6 (3.26)	75 (40.76)	56 (30.43)	27 (14.67)	26 (14.13)	89 (48.37)	64 (34.78)	22 (11.96)	9 (4.89)
	No	153 (54.64)	71 (25.36)	31 (11.07)	22 (7.86)	3 (1.07)	156 (55.71)	76 (27.14)	34 (12.14)	14 (5)	167 (59.64)	79 (28.21)	24 (8.57)	10 (3.57)
		**0.030**				**0.008**				0.153				
**Marital Status** ***N*** **(%)**	Single	246 (54.67)	109 (24.22)	55 (12.22)	30 (6.67)	10 (2.22)	231 (51.33)	129 (28.67)	51 (11.3)	39 (8.67)	268 (59.56)	124 (27.56)	43 (9.56)	15 (3.33)
	Married	110 (43.48)	75 (29.64)	38 (15.02)	26 (10.28)	4 (1.58)	121 (47.83)	73 (28.85)	41 (16.2)	18 (7.11)	133 (52.57)	80 (31.62)	29 (11.46)	11 (4.35)
		**0.045**				0.282				0.343				
**Do you have children** ***N*** **(%)**	Yes	235 (55.95)	98 (23.33)	49 (11.67)	29 (6.9)	9 (2.14)	221 (52.62)	120 (28.57)	43 (10.24)	36 (8.57)	252 (60)	107 (25.48)	46 (10.95)	15 (3.57)
	No	131 (43.67)	90 (30)	45 (15)	28 (9.33)	6 (2)	142 (47.33)	85 (28.33)	49 (16.33)	24 (8)	159 (53)	100 (33.33)	29 (9.67)	12 (4)
		**0.027**				0.106				0.131				
**Years of professional experience** ***N*** **(%)**	1 to 3	32 (33.33)	33 (34.38)	18 (18.75)	12 (12.5)	1 (1.04)	38 (39.58)	31 (32.29)	14 (14.58)	13 (13.54)	44 (45.83)	35 (36.46)	14 (14.58)	3 (3.13)
	4 to 6	47 (41.96)	33 (29.46)	19 (16.96)	8 (7.14)	5 (4.46)	50 (44.64)	26 (23.21)	24 (21.43)	12 (10.71)	54 (48.21)	37 (33.04)	13 (11.61)	8 (7.14)
	7 to 9	45 (48.91)	17 (18.48)	20 (21.74)	9 (9.78)	1 (1.09)	41 (44.57)	30 (32.61)	12 (13.04)	9 (9.78)	46 (50)	31 (33.7)	10 (10.87)	5 (5.43)
	>10	242 (57.62)	105 (25)	37 (8.81)	28 (6.67)	8 (1.9)	234 (55.71)	118 (28.1)	42 (10)	26 (6.19)	267 (63.57)	104 (24.76)	38 (9.05)	11 (2.62)
		**<.0001**				**0.005**				**0.011**				
**What is your working position** ***N*** **(%)**	Front line	84 (42)	58 (29)	32 (16)	20 (10)	6 (3)	89 (44.5)	54 (27)	29 (14.5)	28 (14)	91 (45.5)	67 (33.5)	28 (14)	14 (7)
	Second line	282 (54.23)	130 (25)	62 (11.92)	37 (7.12)	9 (1.73)	274 (52.69)	151 (29.04)	63 (12.12)	32 (6.15)	320 (61.54)	140 (26.92)	47 (9.04)	13 (2.5)
		**0.048**				**0.004**				**0.0002**				

*The bold values are the p-values indicating that the test is significant at P < 0.05*.

The Chi-square test for the scores of these instruments with demographic variables showed that depression, anxiety, and insomnia were correlated significantly according to the age groups with *P* < 0.0001 for all the three tools, years of professional experience was associated with p < 0.001, *P* = 0.005, and *p* = 0.011 respectively; and the working position was associated with *P* = 0.048, *p* = 0.004, and *p* = 0.0002, respectively. Depression and anxiety were significantly related to being Saudi vs. non-Saudi with *p* < 0.0001 and *p* < 0.0005, respectively. Working from home in isolation or not working from home was associated with depression and anxiety with *p* = 0.03 and *p* = 0.008, respectively, whereas insomnia was not significantly related to these variables. Depression was significantly related to working in the MOH or not with *p* = 0.046, marital status with *p* = 0.045, and having children with *p* = 0.027. Only anxiety was related to the professional title with *P* = 0.004.

### Associated Factors of Mental Health Outcomes

The multivariate logistic regression analysis was used with age, gender, and other selected variables from the bivariate analysis to identify the associated risk factors. The results showed that being a non-Saudi and second-line HCW had a lower risks of developing depression (OR, 0.6; 95% CI, 0.38–0.96, *p* = 0.032), and (OR, 0.65; 95% CI, 0.44–0.97, *p* = 0.034), respectively. Being a female and living with family and working from an isolated room was a risk factor for depression (OR, 1.58; 95% CI, 1.03–2.43, *p* = 0.038), and (OR, 1.55; 95% CI, 0.95–2.51, *p* = 0.02), respectively. For developing anxiety nurses (OR, 0.47; 95% CI, 0.23–0.94, *p* = 0.017), and second-line HCWs were at lower risk (OR, 0.55; 95% CI, 0.36–0.85, *p* = 0.001). The second line HCW was at low risk (OR, 0.55; 95% CI, 0.39–0.77, *p* = 0.001) for developing insomnia (Table 3).

**Table 3:** Associated factors for mental health outcomes identified by multivariable regression analysis.

**Variable**	**OR (CI)**	***P*-value**
**PHQ-9 depression symptoms**		
Gender-Male	1	–
Gender-Female	1.49 (0.99–2.24)	0.056
Age-18–25	1	–
Age-26–30	0.95 (0.38–2.33)	0.457
Age-31–40	0.83 (0.3–2.29)	0.858
Age->40	0.54 (0.17–1.65)	0.118
Nationality-Saudi	1	–
**Nationality-non-Saudi**	**0.6 (0.38–0.96)**	**0.032**
Experience-1–3	1.18 (0.55–2.55)	0.805
Experience-4–6	1.23 (0.63–2.4)	0.91
Experience-7–9	1.7 (0.95–3.06)	0.133
Experience->10	1	–
Belongs to Ministry-Yes	1.36 (0.86-2.16)	0.19
Belongs to Ministry-No	1	–
Marital status-Married	1 (0.55–1.82)	0.868
Marital status-Other	0.87 (0.23–3.27)	0.839
Marital status-Single	1	–
**Living with your family-Yes, isolated in a room**	**1.55 (0.95**–**2.51)**	**0.02**
Living with your family-Yes, working from home	0.92 (0.56–1.52)	0.158
Living with your family-No	1	–
Children-Yes	1.2 (0.66–2.18)	0.555
Children-No	1	–
**Work position-second line**	**0.65 (0.44**–**0.97)**	**0.034**
Work position-Front line	1	–
**GAD-7 anxiety**		
Gender-Male	1	–
**Gender-female**	**1.58 (1.03-2.43)**	**0.038**
Age-18–25	1	–
Age-26–30	1.01 (0.38–2.64)	0.702
Age-31–40	1.13 (0.4–3.23)	0.302
Age->40	0.64 (0.2–2.06)	0.173
Nationality-Saudi	1	–
Nationality-Non-Saudi	0.81 (0.48–1.37)	0.432
**Profession-nurse**	**0.47 (0.23**–**0.94)**	**0.017**
Profession-Pharmacist	0.78 (0.42–1.45)	0.988
Profession-Physician	1.02 (0.56–1.87)	0.116
Profession-Other	1	–
Experience-1–3	1.06 (0.48–2.37)	0.704
Experience-4–6	1.55 (0.79–3.04)	0.159
Experience-7–9	1.13 (0.6–2.12)	0.888
Experience->10	1	–
Living with your family-Yes, isolated in a room	1.37 (0.82–2.3)	0.088
Living with your family-Yes, working from home	0.9 (0.54–1.5)	0.212
Living with your family-No	1	–
**Work position-second line**	**0.55 (0.36**–**0.85)**	**0.007**
Work position-Front line	1	–
**ISI-Insomnia symptoms**		
Gender-Male	1	–
Gender-Female	1.18 (0.86–1.63)	0.316
Age-18–25	1	–
Age-26–30	1.83 (0.79–4.22)	0.1
Age-31–40	1.58 (0.64–3.9)	0.322
Age->40	1.11 (0.42–2.93)	0.374
Experience-1–3	1.61 (0.82–3.16)	0.325
Experience-4–6	1.32 (0.74–2.33)	0.971
Experience-7–9	1.37 (0.82–2.3)	0.79
Experience->10	1	–
**Work position-second line**	**0.55 (0.39**–**0.77)**	**0.001**
Work position-Front line	1	–

*The bold values are indicates the Odds ratio, confidence interval and P < 0.05*.

## Discussion

This cross-sectional study investigates the psychological state of HCWs from tertiary care MNGHA Centers and the MOH Centers from various regions in Saudi Arabia. It was observed that the symptoms of depression, anxiety, and insomnia were reported only in a small percentage of HCWs in our study. The median total score for depression and anxiety was 4 (IQR-2.00–9.00, IQR-1.00–8.00, respectively) and insomnia 6 (IQR-2.00–12.00), indicating minimal to mild symptoms according to the established literature (28–30). In this study, most of the respondents were second-line HCWs (72.22%), who were not in direct contact with treating COVID 19 patients.

Some studies done in KSA, and neighboring countries have reported the psychological distress faced by HCWs during COVID 19 crisis. Our study was conducted between April and May 2020; out of 720 participants' physicians, nurses, and pharmacists constituted 87% of the respondents, with only one-third of the participants working as frontline HCWs. The depression in HCWs in our study (23% with moderate to severe depression) was lesser compared to AlAteeq et al. (20) (30.3% with moderate to severe depression) and Almater et al. (21) (29% with severe depression). The rate of anxiety reported by Alzaid et al. was 21.1%, which is similar to our study (21.1% with moderate to severe anxiety); however, Almater et al. (21) and Temsah et al. (26) reported a higher rate of anxiety at 28.9 and 38.3%, respectively, in HCWs. The rate of insomnia reported in our study (14% moderate to severe insomnia) is close to that reported by Almater et al. (21) of 15% among HCWs. AlHanawi et al. (27) reported that 23.4% of HCWs were suffering from severe distress. Alenazi et al. (24) reported 32.3% of HCWs with high anxiety levels. Al Sulais et al. (25) reported two-thirds of the physicians felt worried and isolated, while half of the physicians reported fear. Naser et al. (32) studied HCWs in Jordan using the PHQ-9 and GAD-7 scales, where they reported depression in 21.2% and anxiety in 11.3% HCWs, which is close to that reported in our study. A study done by Aoun et al. in various countries worldwide included Canada, North and South America, UK, Middle East, Europe, and Africa, reported moderate to severe anxiety in 23.6% HCWs using GAD-7 which is close to our study. They used the PHQ-2 instrument and reported depression in 27.4% of the participants (33). A study done in Oman on HCWs used the GAD-7 and Perceived Stress Scale(PSS) reported 26% with moderate to severe anxiety and mean score for PSS was 24 (34). A study done in Eygpt and city of Madinah from KSA used the Arabic version of DASS 21 scale and reported that HCWs in Egypt and KSA 29.6% had severe to very severe depression, 27.0% had severe to very severe anxiety,19.3% had severe to very severe stress and 37.3% experienced inadequate sleeping (35). Most of these studies were conducted during the same period between March to May 2020. It is noted that the use of different evaluation tools and the use of different classifications even if the same scale was used, lead to very different figures being reported for the prevalence rate of mental distress.

The rate of psychological distress amongst HCWs worldwide is much higher than reported in some Middle East countries. A study done in turkey between April and May 2020 reported a prevalence rate of depression (77.6%), anxiety (60.2%), and insomnia (50.4%)in HCWs (36). A study done in New York HCWs in April 2020 reported symptoms of depression (PHQ-9) in 48% and anxiety (GAD-7) in 33% of respondents (4). According to a recent study done in China by Lai et al. (37) between January and Febuary 2020, it was reported that there were high rates of depression 50.4% (PHQ-9≥5) for depression, 44.6% (GAD-7≥5) for anxiety, and 34.0% (ISI≥8) for insomnia. According to a review, 36.1% of Chinese HCWs during the COVID-19 experienced symptoms of insomnia (38). Zhang et al. (39) study done between January and Febuary 2020 reported a prevalence of 50.7% (PHQ-9≥5), 44.7% (GAD-7 ≥ 5), 36.1 % (ISI ≥ 8), respectively. Tian et al. (40) study done in April 2020 reported a prevalence of depression, anxiety, insomnia, and perceived stress of 45.6% (PHQ-9 ≥ 5), 20.7% (GAD-7 ≥ 5), 27.0% (ISI ≥ 8). Rossi et al. (41) study was done in Italy in March 2020 reported a prevalence of 24.7% (PHQ-9≥15).

Most of the studies done worldwide were conducted between March to April when there was a peak in the COVID-19 infections in their respective countries. Except for Chinese studies conducted from January to February, during this period they had highest infection rate. The prevalence of depression in our study (23%) was similar to Rossi et al. (24.7%). The Turkish HCWs had the highest depression (77.6%), followed by the Chinese HCWs, with almost half (45.6–50%) reporting depression. A similar proportion of HCWs (48%) in New York City reported depression. The proportion of HCWs reporting anxiety in our study (21%) was similar to a Chinese study by Tian et al. (20.7%). In most other studies, the proportion of HCWs reporting anxiety was two times more than our study (60.2–44.7%). The lowest proportion of HCWs in our study (14%) reported insomnia compared to all other studies (15–50.4%).

In our study, females did not suffer from more psychological distress than males. Nevertheless, females were significantly more anxious than males. Our finding is similar to those reported in other studies where male respondents were significantly less predicted to have generalized anxiety than females (20, 22, 37). A Korean study reported that females had a higher prevalence of psychiatric disorders than males.However, compared to the control group, the odds ratios for psychiatric disorders were higher in male HCWs than in females (42). A Chinese study reported that female gender was positively associated with anxiety in frontline HCWs (43).

We observed a significant relationship between different age groups and suffering from depression, anxiety, and insomnia, but age was not a significant predictor of psychological distress. On the other hand, two Saudi studies reported that HCW between 30–39 and 40–49 years had significantly higher depression scores (20, 27). A Turkish study reported higher depression and anxiety scores in the 26–30 age groups than in other age groups (36). In China, it was reported that HCW in the 31–40-year age groups were more worried about infecting their families compared with other groups (44). Additionally a Chinese study found that age was negatively associated with depression, anxiety and insomnia (43).

This study reported a significant association between developing depression and anxiety with the nationality of HCWs (Saudi vs. non-Saudi). Contrary to other studies, our study did not find a significant difference amongst the nurse, physician, and pharmacist with other professional HCWs with depression and insomnia (37). It was observed in this study that the nurses were more anxious than other professionals. Similar findings were reported in the study done by Alateeq et al. (20). A study by Aoun et al. (33) also reported significantly higher levels of anxiety in nurses compared to physicians. In other studies, it was observed that female nurses with close contact with COVID-19 patients appeared to suffer from the highest mental health risks (3, 36, 38). A Chinese study had reported that being a physician was a protective factor against insomnia (37).

We found a significant association between depression and anxiety, with residing with family. Additionally, depression was also significantly associated with having children. Similar findings were reported by Alzaid et al. (22) where living with family members was predicted to increase anxiety. A Chinese study found having ≥2 children as a risk factor for developing depression in HCWs (45). A Saudi study found living with an elderly to be significantly related to depression in physicians (21). Our study observed that living with family and working from home in an isolated, separate room was a predictor of developing depression. Similar findings were reported in a Chinese study were nurses working in isolated wards were more prone to depressive symptoms (38, 46).

We also found a significant association between the number of years of professional experience and depression, anxiety, and insomnia. However, this was not found to be a risk factor for developing mental distress. A review reported that as the professional experience increased, HCWs were less likely to develop psychiatric disorders (38). Contrary to this, a Turkish study observed depression and anxiety scores in participants working for 10 years or more were significantly higher than those working for <10 years (36). In our study, we did not find any association between psychological distress and location, unlike other studies where they found HCWs living in the Central region (Riyadh) reported higher scores for anxiety and depression compared to other regions (20). Similar results were reported from Chinese studies where HCWs in Wuhan reported significantly more mental distress than outside (37).

We observed that working as a frontline HCW was a predictor for depression, anxiety, and insomnia. Other studies were done in KSA also reported that frontline female ophthalmologists suffered from depression, and in general, ophthalmologists showed significant signs of anxiety and insomnia (21, 27). This finding is in line with reports from the literature where the frontline workers are in constant contact with COVID 19 infected patients; hence they suffer from more significant psychological distress (38, 47, 48). According to a review, inconsistent results were reported for front-line health workers. One study reported that they are at higher risk than colleagues, and other studies suggesting no vital difference in stress for the department (49). A Turkish study reported no difference in terms of depression or anxiety symptoms in front line workers than second-line workers (36). A study was done by Aoun et al. (33) also reported that working as a nurse or dentist outside of the middle east was associated with moderate to severe anxiety.

We noted one crucial factor that was not studied in most of the studies that were the workload of the HCWs during the crisis.A study by Zhou et al. (43) reported that daily working hours were positively related to psychological disorders. A study by Mo et al. (50) reported that long work hours were significantly related to stress and anxiety. An Iranian study also demonstrated that nurses faced more mental stress than other HCWs due to increased workload and shift timings (51). A systemic review of studies on COVID 19 also highlighted that little support and high workload were well-known risk factors for mental health problems among HCWs in times of crisis (52). In contrast to this, a study done in KSA by Alzaid et al. (22) reported that most of HCWs were satisfied with their shift arrangement and most of them never thought of resigning. Another study by Alsulais et al. (25) illustrated only 16% participants had reported an increase in their workload.

In general, in pandemic HCWs are expected to experience psychological disorders due to home quarantine and social isolation, disrupted work routines, closure of public, and private institutions, obsession with cleanliness, amongst a few reasons. Nevertheless, our study reported a low level of mental stress attributed to the Saudi Governments immediate measure to contain the pandemic. Presently, the mortality rate in Saudi Arabia is low; as of 3 June 2020, the mortality rate reported was 0.9% for the severely infected and 0.2% for the mild and asymptomatic cases (53). Low mortality can be credited to the severe preventive measures taken by the Kingdom of Saudi Arabia since March 2020, some of which included travel restrictions on all domestic and international flights, the lockdown of cities, total or partial curfews, and the closing of mosques, shopping malls, and recreation centers to limit the spread of infection (54). The government took immediate steps to establish mental health support programs. Institutional level initiatives were taken to meet the growing need for psychological care for HCWs concentrating on four areas; education, therapy, awareness, and prevention. The ministry of health established a hotline to support HCWs to address their concerns. Specialized clinics were dedicated to employees to meet the growing demand for mental care and avoid burnout or mental breakdown. The DA'EM web-based wellness program was established 24 h, which was anonymous to provide psychological support to HCWs across the Kingdom (54). The steps taken by the Kingdom of Saudi Arabia are in line with some strategies suggested in the literature to provide support to HCWs, which included psychological intervention support teams, psychological counseling, availability of helpline, online platforms for medical assistance (3, 48).

### Limitations

The majority of the respondents in our study were from the central region and were second-line HCWs. We did not explore the common risk factors for depression, like a history of depression, comorbidities like chronic diseases, social support, and communication, as this was beyond our study's scope. We administered an online survey, and physician-led psychiatric evaluation was not done; hence, we could not explore if participants were already suffering from a psychiatric disorder. The English version of the instruments used did not undergo the cultural adaptation process since this was beyond our study's scope. This study's results cannot be generalized, as we did not cover all KSA regions and areas.

Furthermore, we were not able to assess the workload and its psychological impact on the HCWs. Personality traits, such as optimism, resilience, and altruism, exercise habits have previously been shown to have positive effects on reducing psychological stress (44, 45). As the HCWs were found to have a better mental state in KSA than to many other studies during this time, further research is required to evaluate organizations' preparedness to deal with pandemics, personality traits, and coping behaviors practiced by HCWs in KSA.

## Conclusion

The symptoms of depression, anxiety, and insomnia were reported in a lower proportion of HCWs, indicating good management of the organizations's health care environment and initiatives. These professionals can work to their full potential and provide maximum support for the front line HCWs and other auxiliary services. In general, the risk factors for depression in HCWs in KSA were nationality (Saudi), living with family, and working from isolated rooms and work positions (frontline). For anxiety, female, nurse, and frontline HCW were risk factors, and only frontline HCW was a risk factor for insomnia.

## Data Availability Statement

The raw data supporting the conclusions of this article will be made available by the authors, without undue reservation.

## Ethics Statement

The studies involving human participants were reviewed and approved by Institutional Review Board, King Abdullah International Medical Research Center (KAIMRC), King Saud Bin Abdul Aziz University for Health Sciences. The patients/participants provided their written informed consent to participate in this study.

## Author Contributions

MA and KS conceived of the study, proposal development, its design and coordination, and acquisition of data. MA participated in writing and critically revising the manuscript. KS and AT participated in data analysis, data interpretation, and writing and critically revising the manuscript. LA and NA participated in survey administration, cleaning data, and critically revising the manuscript. All authors read and approved the final manuscript.

## Conflict of Interest

The authors declare that the research was conducted in the absence of any commercial or financial relationships that could be construed as a potential conflict of interest.
